# MAM domain containing 2 is a potential breast cancer biomarker that exhibits tumour‐suppressive activity

**DOI:** 10.1111/cpr.12883

**Published:** 2020-07-24

**Authors:** Hyeonhee Lee, Bum‐Chan Park, Jong Soon Kang, Yeongmi Cheon, Soojin Lee, Pil Jae Maeng

**Affiliations:** ^1^ Department of Microbiology and Molecular Biology Chungnam National University Daejeon Republic of Korea; ^2^ Y‐Biologics Inc Daejeon Republic of Korea; ^3^ Laboratory Animal Resource Center Korea Research Institute of Bioscience and Biotechnology Cheongju Republic of Korea; ^4^ Gwangju Center Korea Basic Science Institute (KBSI) Gwangju Republic of Korea

**Keywords:** biomarkers, breast cancer, cell proliferation, tumour suppressor

## Abstract

**Objectives:**

The aim of this study was to discover new potential biomarkers of breast cancer and investigate their cellular functions.

**Materials and methods:**

We analysed the gene expression profiles of matched pairs of breast tumour and normal tissues from 24 breast cancer patients. Tetracycline‐inducible MAMDC2 expression system was established and used to evaluate cell proliferation in vitro and in vivo. MAMDC2‐mediated signalling was determined using immunoblot analysis.

**Results:**

We identified MAMDC2 as a down‐regulated gene showing significant prognostic capability. Overexpression of MAMDC2 or treatment with MAMDC2‐containing culture medium significantly inhibited the cell proliferation of T‐47D cells. Furthermore, MAMDC2 expression reduced in vivo growth of T‐47D xenograft tumours. MAMDC2 may exert its growth‐inhibitory functions by attenuating the MAPK signalling pathway.

**Conclusion:**

We report that MAMDC2 has a tumour‐suppressive role and, as a secretory protein, it might be useful as a biomarker for breast cancer treatment.

## Graphical abstract

To discover new potential biomarkers, we analyzed the gene expression profiles of breast tumor tissues and identified MAMDC2 as a down‐regulated gene. MAMDC2 showed strong growth inhibitory activity in estrogen receptor (ER)‐positive breast cancer cell lines. MAMDC2 may exert its tumor suppressive role by attenuating the MAPK signaling pathway as an extracellular regulator. We also found that ER may suppress MAMDC2 expression, suggesting this protein as an important growth regulator in ER+ breast cancer cells.

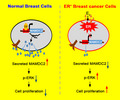

**Abbreviations**
IDC: invasive ductal carcinoma
MAM: meprin/A‐5 protein/receptor protein‐tyrosine phosphatase mu
MAMDC2: MAM domain containing 2
ROC: receiver operator characteristic

## 1 INTRODUCTION

Breast cancer is a leading cause of morbidity and mortality worldwide and accounts for 25% of all cancer cases in women.^1^, ^2^ Over 2 million new cases were diagnosed in 2018 globally, and the incidence rate of breast cancer will likely to rise due to changes in lifestyle and increased life expectancy.^3^ The most common type of breast cancer is invasive ductal carcinoma (IDC), also known as infiltrating ductal carcinoma, and this form constitutes about 80% of all breast cancer diagnoses.^4^ IDC begins in milk ducts, then invades breast tissue and ultimately spreads to lymph nodes.^5^ Among cancers, breast cancer exhibits good survival odds with the 5‐year survival rate at about 90%.^6^ Therefore, early‐stage detection and proper treatment are crucial for long‐term survival for breast cancer patients.^7^

The MAM (meprin/A‐5 protein/ receptor protein‐tyrosine phosphatase mu) domain is a conserved protein domain found in multiple, diverse, cell surface proteins.^8^ One member of the MAM family, MAMDC2 (MAM domain containing 2), is a putative secretory protein that consists of 686 amino acids containing a short N‐terminal signal sequence and four consecutive MAM domains. Several studies reported that *MAMDC2* gene expression is differentially regulated in certain human cancer types, including CML, head and neck squamous cell carcinoma and breast cancer.^9^, ^10^, ^11^ A gene expression analysis reported *MAMDC2* as one of three genes that are correlated with disease‐free survival of breast cancer patients.^12^ Although previous reports have shown that the MAMDC2 expression is associated with various human cancer types, its exact molecular function has not been defined. In the present study, we have demonstrated that MAMDC2 has a growth‐inhibitory function by regulating MAPK signalling pathway.

## 2 MATERIALS AND METHODS

### 2.1 Tissues specimen and expression analysis

Matched pairs of breast cancer and non‐tumour breast tissue were obtained from 24 female patients (average age; 50.5 ± 14.1 years) diagnosed with IDC and undergoing surgical resection. Samples were obtained between April 2008 and December 2009 at the Chungnam National University Hospital (Daejeon, Korea). The study was approved by the Institutional Review Board of the Chungnam National University Hospital and conducted in accordance with the relevant guidelines and regulations. Written informed consent was obtained from all patients for participation in the study.

The differentially regulated genes were selected by statistical analysis using GEO database (http://www.ncbi.nlm.njh.gov/geo): GSE22035, GSE5764, GSE26910 and GSE21422. Gene expression analysis was performed using the Nanostring nCounter system (Nanostring Technologies, USA) according to the manufacturer's instructions. The quantified probe counts were normalized to β‐actin gene. All statistical analyses were performed with Medcalc software (Belgium). *P*‐values less than .05 were considered statistically significant.

### 2.2 Cell culture and RT‐PCR

T‐47D and MDA‐MB‐231 cells were obtained from the Korean Cell Line Bank (Seoul, Korea). All other cell lines were obtained from Y‐Biologics (Daejeon, Korea). MDA‐MB‐231 cells were grown in DMEM media (Welgene, Korea), while the others were maintained in RPMI‐1640 media. RT‐PCR amplification was performed using a monoplex RT‐PCR with 2X TOPsimple™ DyeMix‐multi HOT premix (Enzynomics, Korea).

### 2.3 Plasmids and siRNAs

The full‐length cDNA of human *MAMDC2* (GeneBank accession number NM153267) was obtained from total RNA of MDA‐MB‐231 cells. Then, MAMDC2 gene was amplified and inserted into pcDNA3‐3FLAG^13^ or pEGFP‐N3 (Clontech). For the tet‐inducible system, MAMDC2‐3FLAG was inserted into pcDNA‐4TO (Invitrogen). To generate deletion mutants, the N‐terminal signal peptide fragment (aa 1‐23) was amplified and fused in‐frame with different MAM mutants (D1‐2, aa 24‐329; D2‐3, aa 169‐498; D3‐4, aa 340‐666). For siRNA treatment, siMAMDC2 #1 (CGAGUGAAAGUAAAACCAA) and siMAMDC2 #2 (CUACAUUGGAAGGCUCUAU) were synthesized by Bioneer (Korea).

### 2.4 Antibodies and confocal microscopy

For Western blotting analysis, anti‐FLAG antibody was purchased from Sigma‐Aldrich; anti‐p‐c‐RAF (S338), anti‐ERK1/2 and anti‐p‐ERK1/2 (T202/Y204) from Cell Signaling Technology; anti‐MAMDC2 antibody from Abcam Inc

For the subcellular localization of MAMDC2, HeLa cells were plated on a glass cover slide and transfected with MAMDC2‐EGFP. To visualize mitochondria, pDsRed2‐Mito vector (Takara, Japan) was co‐transfected. For ER, the cells were incubated with monoclonal anti‐KDEL antibody (Enzo Life Sciences), followed by incubation with Alexa Fluor 594‐labelled anti‐mouse antibody (Thermo Fisher Scientific).

### 2.5 In vivo xenograft experiment

Six‐week‐old female NSG mice (The Jackson Laboratory, USA) were acclimated for one week and then ovariectomized. Then, the mice were implanted with 90 days slow‐release oestradiol pellets (0.72 mg/pellet, Innovative Research of America, USA). Then, mice were injected subcutaneously with T‐47D‐Tet‐On‐MAMDC2 or T‐47D‐Tet‐On‐Ctrl cells (4 × 10^7^ cells/mL) and then fed chow containing 625 ppm doxycycline (Harlan, USA). Animal experiments were conducted using protocols approved by the Institutional Animal Care and Use Committee at Korea Research Institute of Bioscience and Biotechnology. Tumour volumes were measured using a Vernier caliper and calculated by the following formula: Tumour volume (mm^3^) = [length (mm) × width (mm) × height (mm)]/2.

### 2.6 Apoptosis assays

For cell cycle analysis, cells were fixed in 70% ethanol overnight. After treatment with RNase I, cells were stained with 5 μg/mL propidium iodide (PI). Annexin V staining was performed using Annexin V‐fluorescein isothiocyanate (FITC) apoptosis detection kit (BD Bioscience) according to the manufacturer's instructions and analysed on a BD FACSCanto II flow cytometer (BD Biosciences). Caspase activity was assessed using a CaspSCREEN^TM^ Flow Cytometric Apoptosis Detection kit (BioVision Inc).

## 3 RESULTS

### 3.1 MAMDC2 is down‐regulated in breast cancer cells

To discover new biomarkers of breast cancer, we obtained matched pairs of breast tissue from tumour and non‐tumour region of 24 female patients diagnosed with IDC (average age; 50.5 ± 14.1 years; Table S1). Then, we selected potential breast cancer biomarkers by analysing microarray data from GEO database and functional annotation through Gene Ontology database. Finally, 24 putative cell surface and secreted proteins were chosen and a total 27 genes, including 3 controls, had their expression profiles analysed using the Nanostring nCounter system (Figure S1). A total of 22 genes were differentially regulated more than 2‐fold, with 9 genes up‐regulated, while 13 were down‐regulated (Table S2).

**FIGURE 1:**
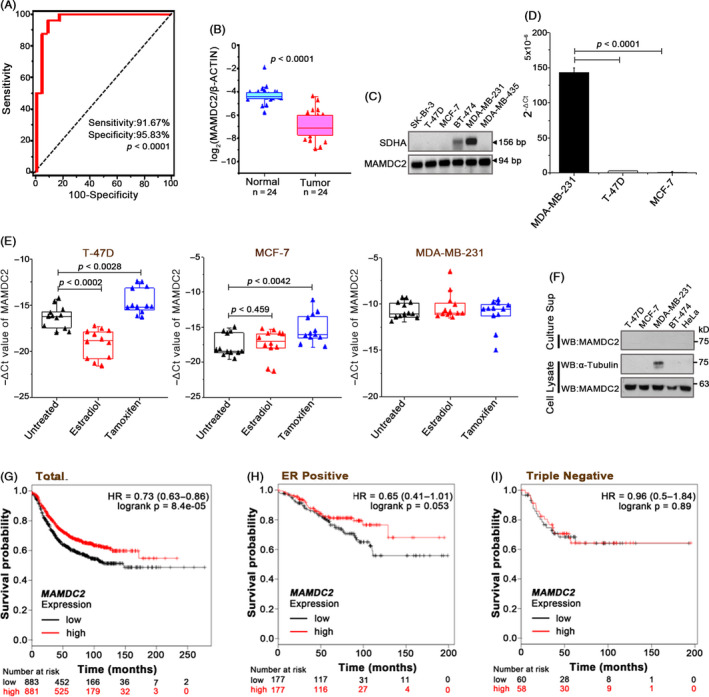
Identification of a novel breast cancer down‐regulated gene, MAMDC2. A, ROC analysis of MAMDC2. B, Comparison graphs of log_2_(MAMDC2/β‐Actin) values between normal and tumour samples. C, MAMDC2 mRNA expression in breast cancer cell lines by RT‐PCR. SDHA, succinate dehydrogenase. D, Quantitative RT‐PCR for endogenous MAMDC2. E, Real‐time RT‐PCR was performed using cells incubated with oestradiol (10 nmol/L) or Tamoxifen (10 μmol/L) for 24 h. The △Ct values of MAMDC2 were normalized to the Ct values for β‐actin. F, Immunoblotting with an anti‐MAMDC2 antibody. G‐I, Association between MAMDC2 expression and overall survival curves of total, ER + subtype, and triple‐negative subtype. Data were retrieved from the KM plotter (http://kmplot.com/analysis/)

Next, we evaluated the utility of these differentially regulated genes for breast cancer diagnosis, using a receiver operator characteristic (ROC) curve analysis. Among the 22 genes, MAM domain containing 2 (MAMDC2) showed a reasonable degree of diagnostic capability as a breast cancer biomarker (*P < *.0001; Figure 1A). The expression level of MAMDC2 was down‐regulated ~5.9‐fold in the breast tumour tissues compared with paired normal tissues (Figure 1B).

To compare the MAMDC2 expression in different breast tumour cell lines, we performed RT‐PCR. Among these, MDA‐MB‐231 expressed the highest level of MAMDC2, while it was undetectable in other cell lines including MCF‐7 and T‐47D cells (Figure 1C), which was confirmed by quantitative real‐time PCR (Figure 1D). To examine the effect of oestrogen receptor (ER) activity on MAMDC2 expression, we incubated cells with oestradiol or tamoxifen under steroid‐free culture condition. While no significant change was found in ER‐negative MDA‐MB‐231 cells, ER inactivation by tamoxifen increased MAMDC expression in both T‐47D and MCF‐7 cells (Figure 1E). Furthermore, MAMDC2 mRNA level decreased by oestradiol treatment in T‐47D cells, suggesting that ER activation is associated in the transcriptional repression of MAMDC2. When we performed immunoblotting with an anti‐MAMDC2 antibody, we could readily observe MAMDC2 protein in MDA‐MB‐231 cell lysates, while none was detected in the culture supernatant (Figure 1F).

Next, in order to evaluate MAMDC2 as a useful biomarker, we tried to detect MAMDC2 protein in human serum or breast tissues. Unfortunately, however, the MAMDC2 protein was not monitored in the human sample due to the lack of specific antibody. Instead, we used the Kaplan‐Meier (KM) plotter database and found a close relation between the MAMDC2 expression level and survival rates of the breast cancer patients. High MAMDC2 expression group showed clearly increased survival curve in total (Figure 1G) and ER‐positive (Figure 1H) patients. Interestingly, however, we could not find any differences in the triple‐negative cases (Figure 1I). Overall, these clinical data may indicate that MAMDC2 is a reliable prognostic biomarker for breast cancer.

### 3.2 MAMDC2 is an *N*‐glycosylated secretory protein

**FIGURE 2:**
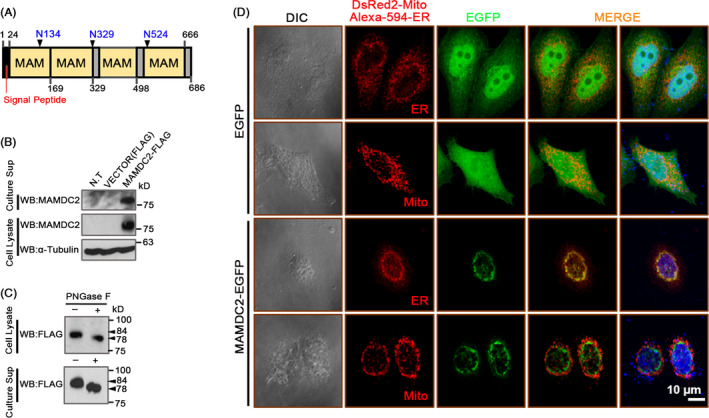
Structure and subcellular localization of human MAMDC2. A, Schematic of predicted structure of human MAMDC2. The predicted N‐linked glycosylation sites are represented as filled‐black triangles. B‐C, Immunoblotting of MAMDC2 in T‐47D cell lysate and culture supernatant. D, HeLa cells were transfected with MAMDC2‐EGFP. If necessary, cells were co‐transfected with pDsRed2‐Mito vector (Mitochondria) or stained with anti‐KDEL antibody (ER)

Although it has not been documented, MAMDC2 was predicted to be a secretory protein, based on the presence of a short N‐terminal signal sequence and absence of any transmembrane domain (Figure 2A). To determine whether this is the case, we transiently transfected pcDNA3‐MAMDC2‐FLAG plasmid into T‐47D cells and monitored MAMDC2 expression by immunoblotting. Interestingly, we observed high amounts of MAMDC2 protein in both cell lysate and the culture supernatant, indicating that it is secreted into the extracellular space (Figure 2B). We next examined the glycosylation status of MAMDC2 protein by treating it with PNGase F. The molecular weight of MAMDC2‐FLAG protein collected from both cell lysate and culture supernatant was reduced after incubation with PNGase F, demonstrating that both cellular and secreted MAMDC2 proteins are N‐glycosylated (Figure 2C).

In order to determine the intracellular localization of MAMDC2 proteins, we transfected pEGFP‐MAMDC2 construct into HeLa cells. In contrast to EGFP which is dispersed throughout the cytosol, EGFP‐conjugated MAMDC2 substantially co‐localized with ER‐Tracker, but not with Mito‐Tracker (Figure 2D). This suggests that MAMDC2 protein is synthesized in the ER and released from the cells via a secretory pathway.

### 3.3 Effect of MAMDC2 overexpression on cell proliferation

**FIGURE 3:**
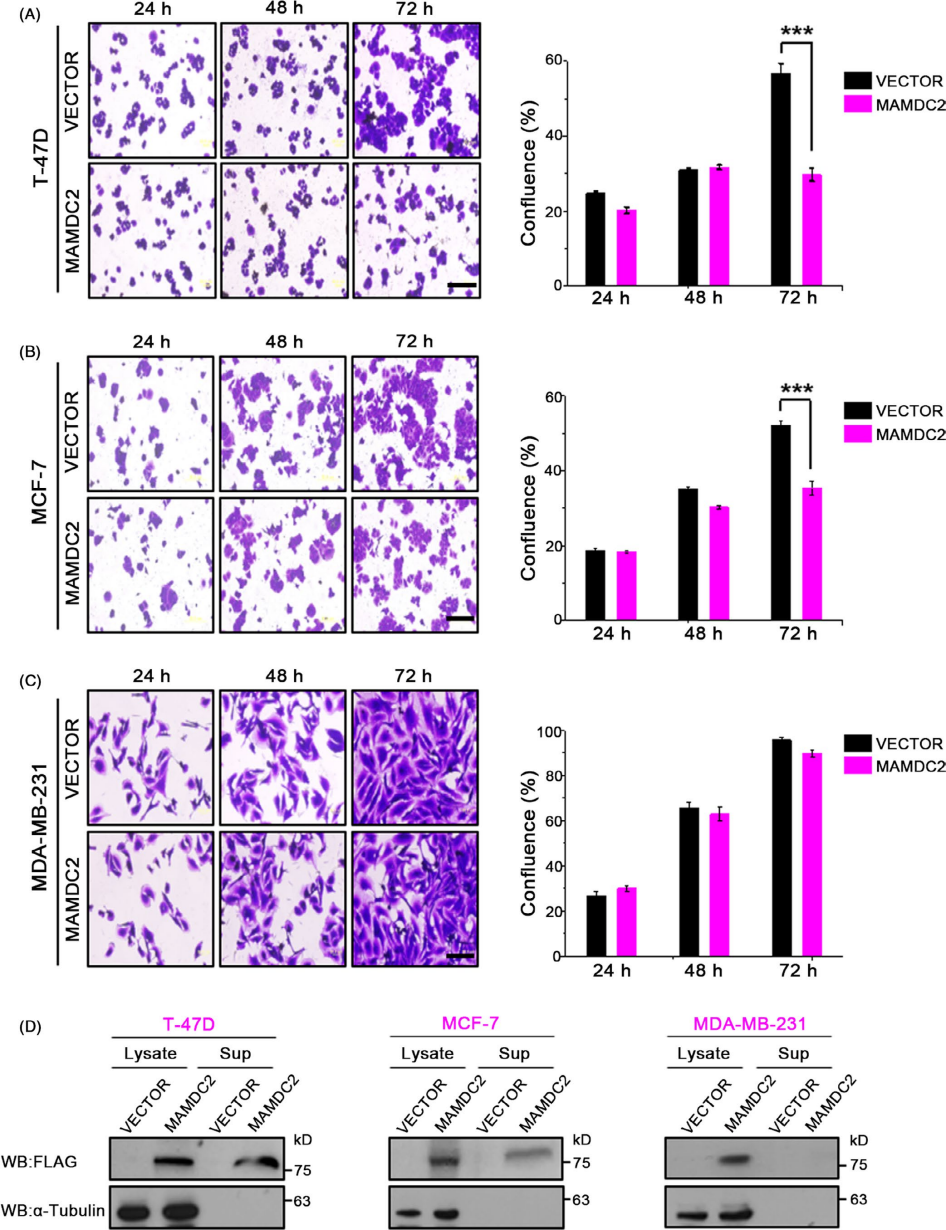
Effect of MAMDC2 on cell proliferation. T‐47D (A), MCF‐7 (B) and MDA‐MB‐231 cells (C) were plated in 6‐well plates and transfected with pcDNA3‐MAMDC2‐FLAG. Then, cell images were captured after crystal violet staining (left). Confluence was estimated by measurement of the stained area of each well using ImageJ software (right). ***P < .0001. Scale bar = 200 μm. (D) Immunoblotting of MAMDC2‐FLAG‐transfected cells

Next, we tested whether overexpression would have any influence on cell proliferation, using T‐47D, MDA‐MB‐231 and HeLa cells. After transfection of pcDNA3‐MAMDC2‐FLAG, cells were stained with crystal violet solution at 24‐hours intervals for 3 days. Cell images were captured, and their confluence estimated using ImageJ software (rsb.info.nih.gov/ij/). MAMDC2 overexpression profoundly inhibited cell proliferation in both T‐47D (Figure 3A) and MCF‐7 cells (Figure 3B), especially by 72 hours post‐transfection. Interestingly, however, we did not observe any difference in the growth of MDA‐MB‐231 cells (Figure 3C). When we performed Western blot analysis with anti‐FLAG antibody, MAMDC2 protein was detected in both cell lysates and culture supernatants of T‐47D and MCF‐7 cells (Figure 3D). In contrast, this protein was only detected in the cell lysates of MDA‐MB‐231, but not in the culture supernatant. These results strongly suggest that the intracellular expression of MAMDC2 is not enough to inhibit cell growth, and therefore, it is likely that MAMDC2 functions as an extracellular regulator of cell proliferation.

### 3.4 The *N*‐terminal region of MAMDC2 is important for its cell growth‐inhibitory activity

**FIGURE 4:**
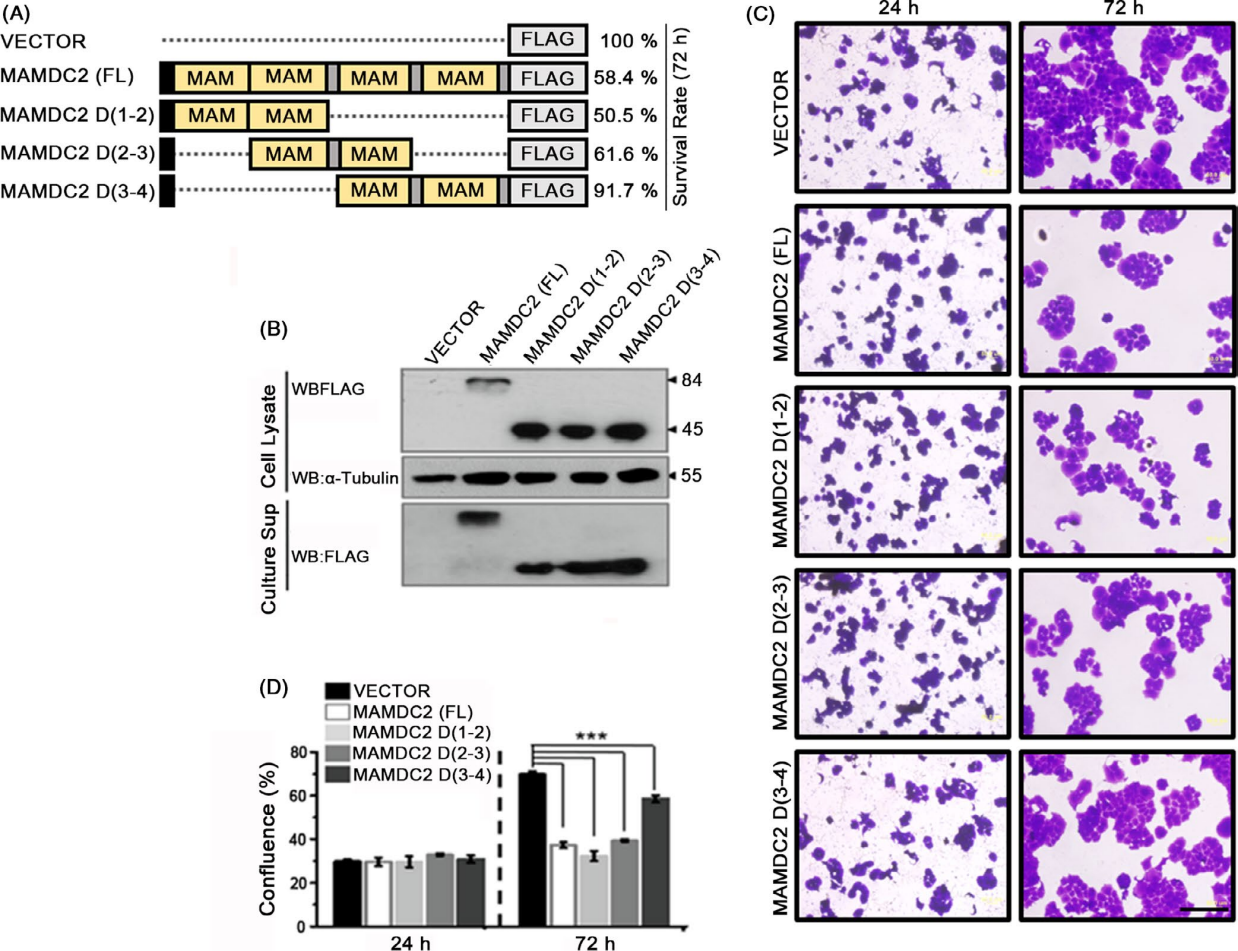
Determination of essential domains of MAMDC2 for anti‐cancer activity. A, Schematic representation of expression vectors for full‐length (FL) and deletion constructs. B, Protein expression of different MAMDC2 deletion mutants. C, Images of the T‐47D cells expressing different MAMDC2 constructs. Scale bar = 200 μm. D, Relative confluence was estimated by measurement of the stained area of (C) using ImageJ. ***P < .0001

To determine which regions are critical for the cell growth‐suppressive function, we generated several deletion mutant constructs of MAMDC2 using pcDNA3‐MAMDC2‐FLAG plasmid. Because this protein contains four sequential MAM domains, that is D1, D2, D3 and D4 from the N‐terminus, we made three truncated versions of MAMDC2 each of which contained two MAM domains, that is D1‐2, D2‐3 and D3‐4 (Figure 4A). Then, their expression was confirmed in both cell lysate and culture supernatant (Figure 4B). When transfected into T‐47D cells, two MAMDC2 variants, D1‐2 and D2‐3, exhibited inhibitory effects on the growth of T‐47D cells that were similar to the wild‐type MAMDC2 (Figure 4C,D). Taken together, we propose that the second MAM domain from the N‐terminus, D2, may have a critical role in mediating cell growth inhibition.

### 3.5 MAMDC2 overexpression induced cell death in T‐47D cells

**FIGURE 5:**
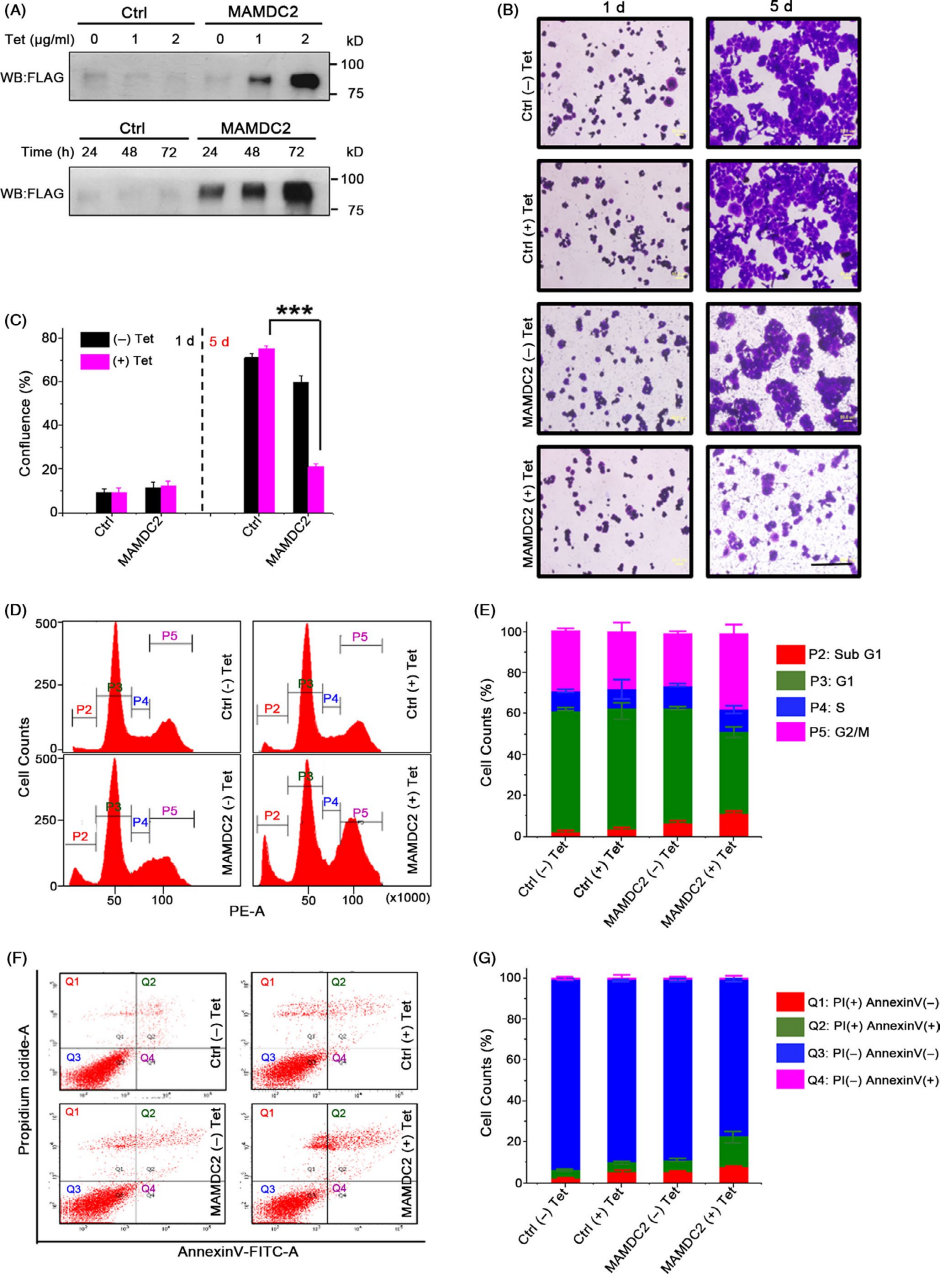
MAMDC2‐mediated cell death in T‐47D‐MAMDC2 stable cells. A, Culture supernatants of T‐47D‐Tet‐On‐MAMDC2 cells were collected after incubation with tetracycline for 48 h. Otherwise, cells were incubated with 2 μg/mL tetracycline for 3 days. (B,C) Images of Tet‐On‐MAMDC2 and Tet‐On‐Ctrl cells from day 1 and day 5 cultures. Scale bar = 200 μm. ***P < .0001. (D,E) Cell cycle analysis using cells incubated with 2 μg/mL tetracycline for 48 h. (F,G) Annexin V/PI staining of MAMDC2‐expressing cells. The error bars represent the standard deviation

Since we failed to obtain MAMDC2‐expressing T‐47D stable cells, we instead established a tetracycline (tet)‐inducible MAMDC2‐FLAG expression system in T‐47D cells (Tet‐On‐MAMDC2) along with vector‐transfected control cells (Tet‐On‐Ctrl). The amount of secreted MAMDC2 increased in proportion to the concentration of tetracycline and gradually accumulated in culture media in a time‐dependent manner (Figure 5A). Next, we monitored the cell proliferation kinetics of these stable cells after 5‐day incubation with tetracycline (2 μg/mL). Cell viability of Tet‐On‐MAMDC2 was dramatically reduced by ~70% upon exposure to tetracycline (Figure 5B,C).

Cell cycle analysis by flow cytometry was performed after Tet‐On‐MAMDC2 cells were incubated with or without tetracycline for 48 hours. The sub‐G1 population significantly increased in Tet‐induced MAMDC2‐expressing cells (Figure 5D,E). In Annexin V staining assay, end‐stage apoptotic cells (both Annexin V and PI positive, Q2) highly increased in these cells (Figure 5F,G). Finally, in vivo caspase activation was assessed using a CaspSCREEN™ Flow Cytometric Apoptosis Detection kit. The apoptotic cell population increased in MAMDC2‐expressing cells (Figure S2), suggesting that MAMDC2 overexpression induces apoptosis in T‐47D cells.

### 3.6 In vivo tumour growth in MAMDC2 xenograft model

**FIGURE 6:**
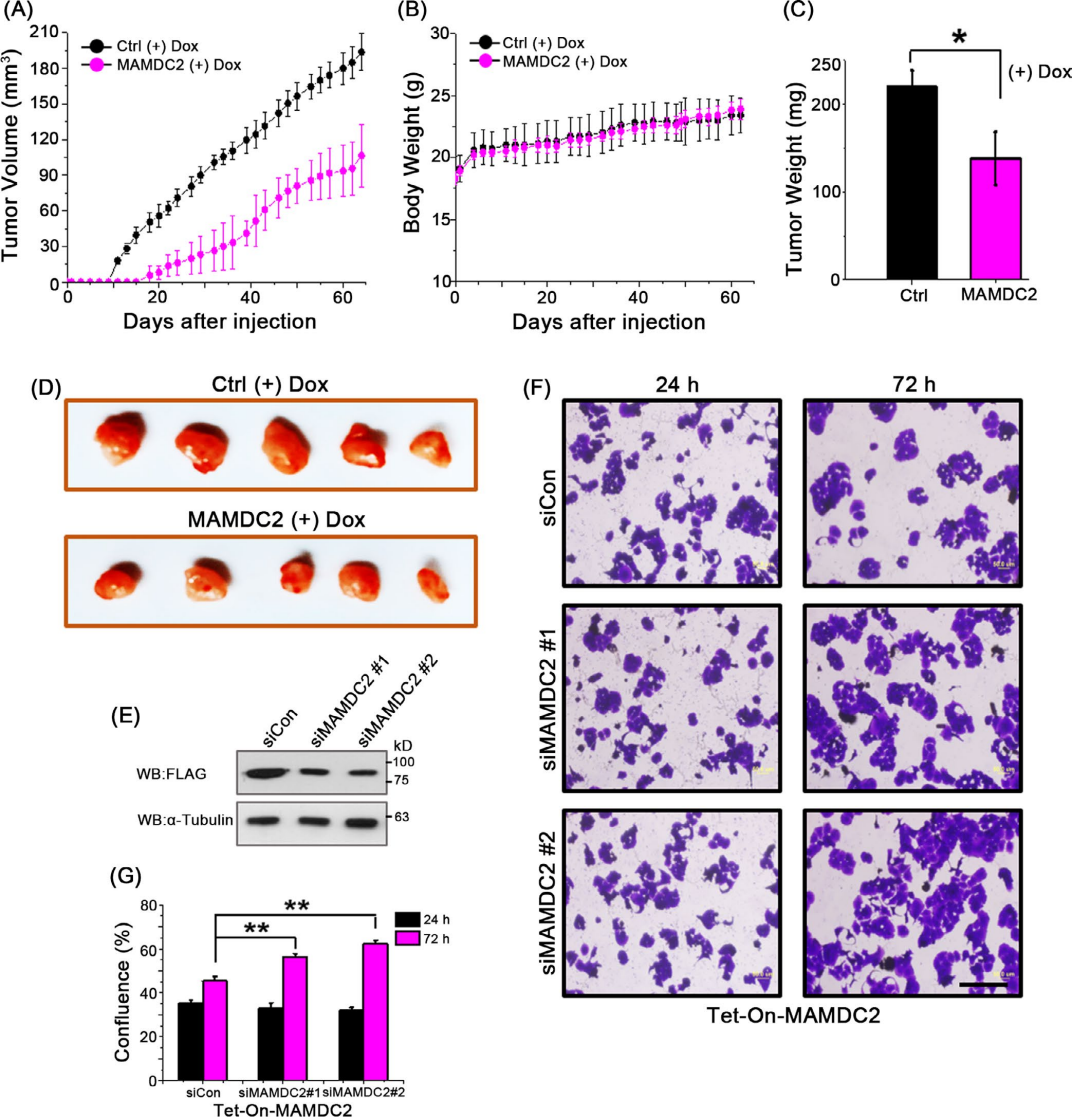
In vivo xenograft experiment. A, Tumour volumes were measured after ovariectomized female NSG mice were injected with Tet‐On‐MAMDC2 cells (n = 5). B, Mean body weights of mice in each group. (C,D) On day 70 after implantation, tumours were collected and weighed. *P < .05. E, For MAMDC2 knockdown, Tet‐On‐MAMDC2 cells were incubated with siRNA duplexes (50 nmol/L) or control siRNA (siCON) for 72 h in the presence of tetracycline. (F,G) Cell viability was examined after incubation with siRNAs for 48 h. **P < .001

To evaluate the effect of MAMDC2 on tumour growth in vivo, Tet‐On‐MAMDC2 cells or Tet‐On‐Ctrl cells were injected into female NOD scid IL2 receptor gamma null (NSG) mice (n = 5). As T‐47D is an ER+ cell line, the xenografted mice also received a slow‐release oestrogen pellet to promote in vivo tumour formation. In addition, MAMDC2 expression was induced by providing food containing doxycycline from day zero. Although the mean body weight of each group did not change significantly, the Tet‐On‐MAMDC2 xenograft mice exhibited tumour regression (Figure 6A,B). Seventy days after tumour cell injection, all mice were sacrificed and tumour xenografts were excised. The average tumour weight of MAMDC2‐expressing xenografts was ~62% of controls (*P* < .05; Figure 6C,D), indicating that the MAMDC2 expression can attenuate in vivo tumour cell proliferation.

Next, we examined the effect of MAMDC2 knockdown on cell proliferation. Because we could not detect endogenous MAMDC2 protein in most cancer cell lines, we performed the experiment using Tet‐On‐MAMDC2 cells. Two siRNAs were selected to repress MAMDC2 expression to about half the level of control siRNA‐treated cells (Figure 6E). While no significant difference was observed in 24‐hours cultures, a clear recovery of growth was observed in siMAMDC2‐treated cells after 72 hours (Figure 6F,G), providing further strong evidence that the expression of MAMDC2 is closely linked with cell viability.

### 3.7 MAMDC2 inactivates RAF‐ERK cascades in T‐47D cells

**FIGURE 7:**
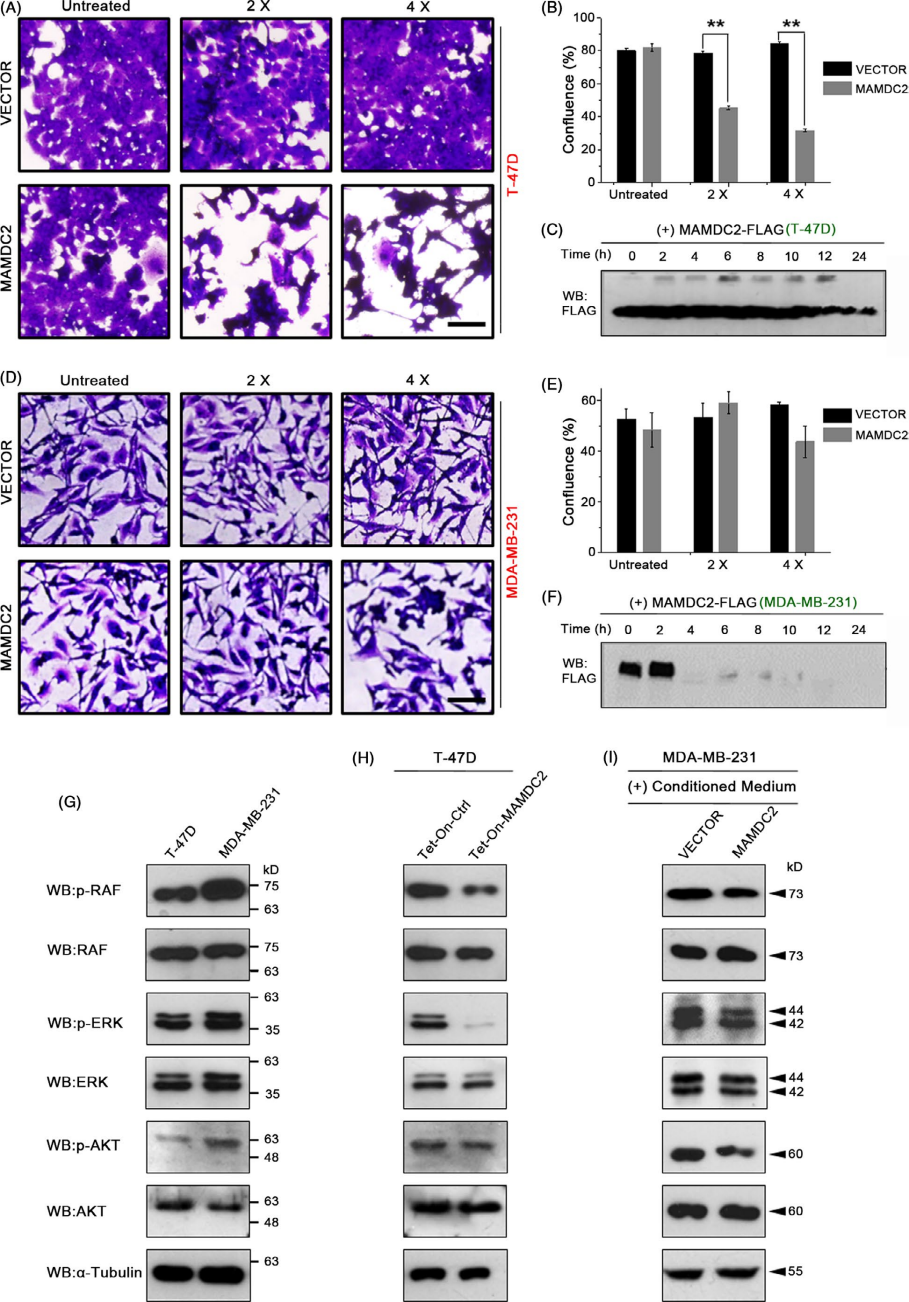
MAMDC2 may inhibit RAS‐ERK pathway. A‐F, The culture media of Tet‐On‐MAMDC2 cells were collected and concentrated 10‐fold. Then, concentrated culture media (10x) was added to cells to final concentrations (0×, 2×, and 4×) for 3 days. Immunoblotting was performed with the culture media of T‐47D (C) and MDA‐MB‐231 cells (F). **P < .001. Scale bar = 200 μm. G, Phosphorylation levels of MAPK components in the normal growth condition (H) Tet‐On‐MAMDC2 or Tet‐On‐Ctrl cells were cultured with tetracycline for 48 h. I, Conditioned media collected from Tet‐On‐MAMDC2 or Tet‐On‐Ctrl cells were added to the culture media (1x) of MDA‐MB‐231 cells for 48 h

Earlier experiments showed that the secreted MAMDC2 was not detected in the culture media of MDA‐MB‐231, despite a strong intracellular expression (Figures 1F and 3D). Furthermore, MDA‐MB‐231 cell growth was not inhibited by MAMDC2 transfection (Figure 3C). To address whether the unresponsiveness of MDA‐MB‐231 cells to MAMDC2 is due to a defect in the secretory pathway, we examined MDA‐MB‐231 cell growth in the presence of conditioned culture media containing MAMDC2 protein. Tet‐On‐MAMDC2 cells were cultured in serum‐free media containing tetracycline for 2 days, and the culture supernatant was concentrated 10‐fold. Then, the concentrated conditioned media were added to T‐47D or MDA‐MB‐231 cultures (final 2× and 4× concentrations). Although T‐47D cells exhibited diminished cell viability (Figure 7A,B), no dramatic changes were found in MDA‐MB‐231 cultures (Figure 7D,E). Interestingly, while MAMDC2 protein remained until 24 hours in the culture media of the T‐47D cells (Figure 7C), it quickly disappeared when added to the MDA‐MB‐231 cells (Figure 7F).

To understand MAMDC2‐mediated signalling, we examined its effect on the phosphorylation of the MAPK pathway using Proteome Profiler™ Human Phospho‐MAPK Array Kit and observed a clear reduction in the levels of phosphorylated AKT and ERK (Figure S3). To verify these results, we performed Western blot analysis for the identified MAPK signalling components. In normal culture condition, the phosphorylation levels of RAF1, ERK1/2 and AKT1 were higher in MDA‐MB‐231 cells compared with those in T‐47D cells (Figure 7G), which is possibly related to the aggressive behaviour of the MDA‐MB‐231 cell line.

Next, we examined the activation status of MAPKs in Tet‐On‐MAMDC2 after 48‐h tetracycline treatment. The phosphorylation levels of RAF1 and ERK1/2 were dramatically decreased in Tet‐On‐MAMDC2 compared with control cells (Figure 7H). Interestingly, phosphorylation of RAF1 and ERK1/2 was not changed in MDA‐MB‐231 cells after treatment with MAMDC2‐containing conditioned media for 48 hours (Figure 7I). Collectively, our results suggest that extracellular MAMDC2 may inhibit cell growth by regulating the RAF‐ERK pathway, which is inoperative in MDA‐MB‐231 cells.

## 4 DISCUSSION

Breast cancer is a highly heterogeneous disease exhibiting diverse clinical features.^14^ Although different studies classify breast cancer cell lines into different categories, the most general subtyping is based on the expression of three immunohistochemistry markers, ER, progesterone receptor (PR) and the human epidermal growth factor receptor 2 (HER2).^15^ Although ER‐positive tumours are very common, accounting for 70 to 80% of all breast cancer cases, only about 30% of cell lines are ER+, because ER‐negative cells are more likely to be established.^16^ Despites the broad phenotypic spectrum of breast cancers, three cell lines (MCF7, T‐47D and MDA‐MB‐231) account for more than two‐thirds of breast cancer studies.^14^ T‐47D, which is often categorized as ‘ER+ luminal subtype’, exhibits low‐levels of genomic abnormalities.^17^ In contrast, MDA‐MB‐231 is a member of the triple‐negative cells that lack all three receptors (ER, PR and HER2) showing high rates of metastasis.

To our surprise, the obvious tumour‐inhibitory function of MAMDC2 shown in T‐47D and MCF‐7 cells was completely impaired in MDA‐MB‐231 cells. Furthermore, we could not detect secreted MAMDC2 protein from MDA‐MB‐231 cells (Figure 3D). While T‐47D is considered as non‐aggressive cell line, MDA‐MB‐231 have a highly aggressive and invasive phenotype that can easily invade extracellular matrix with high metalloproteinase activities.^18^ In fact, the MAMDC2 protein was quickly degraded in the MDA‐MB‐231 culture media (Figure 7F). Therefore, the resistance of MDA‐MB‐231 cells to the MAMDC2‐mediated growth control is related to the rapid degradation of extracellular MAMDC2 protein by metalloproteinases.

The KM plotter analysis revealed that MAMDC2 expression level is closely associated with the survival rates of the breast cancer patients in the ER+ group (Figure 1H), which was completely abrogated in the triple‐negative subtype (Figure 1I). Furthermore, when we analysed, the MAMDC2 expression using two GEO profiles, that in aggressive breast tumour subtypes, was relatively higher than non‐aggressive or less aggressive subtypes (Figure S4). These data may demonstrate that the highly aggressive breast tumour cells are no longer influenced by MAMDC2 expression. Unlike in MDA‐BD‐231 cells, ER signalling appears to negatively regulate MAMDC2 gene expression in T‐47D and MCF‐7 cells (Figure 1E). Since MAMDC2 exerts strong growth‐inhibitory effects on these ER+ cells, ER activation could be more important in these cells to suppress MAMDC2 gene expression. On the contrary, we found that addition of oestradiol did not affect the growth‐inhibitory function of MAMDC2 even in ER+ cells (Figure S5), suggesting that ER signalling is not closely associated with the MAMDC2‐mediated growth control. Collectively, we report that MAMDC2 is a novel tumour inhibitor gene that can be used a prognostic marker for the ER‐positive breast tumour.

## CONFLICT OF INTEREST

The authors declare that they do not have any conflicts of interest with the contents presented in this study.

## AUTHOR CONTRIBUTIONS

All authors participated in study design, data interpretation, and analysis and manuscript review. PJM conceptualized and acquired funding. HL involved in investigation and validation. YC visualized the study. BCP provided the resources. SJ and PJM supervised the study. SL wrote the manuscript.

## Supporting information

Fig S1

Fig S2

Fig S3

Fig S4

Fig S5

Table S1

Table S2

## Data Availability

The data that support the findings of this study are available from the corresponding author upon reasonable request.
