# Supplementary figures and images for: MAM domain containing 2 is a potential breast cancer biomarker that exhibits tumour‐suppressive activity
Source: Cell Prolif. 2020 Jul 24;53(9):e12883. doi: 10.1111/cpr.12883 (PMC7507446; doi:10.1111/cpr.12883)

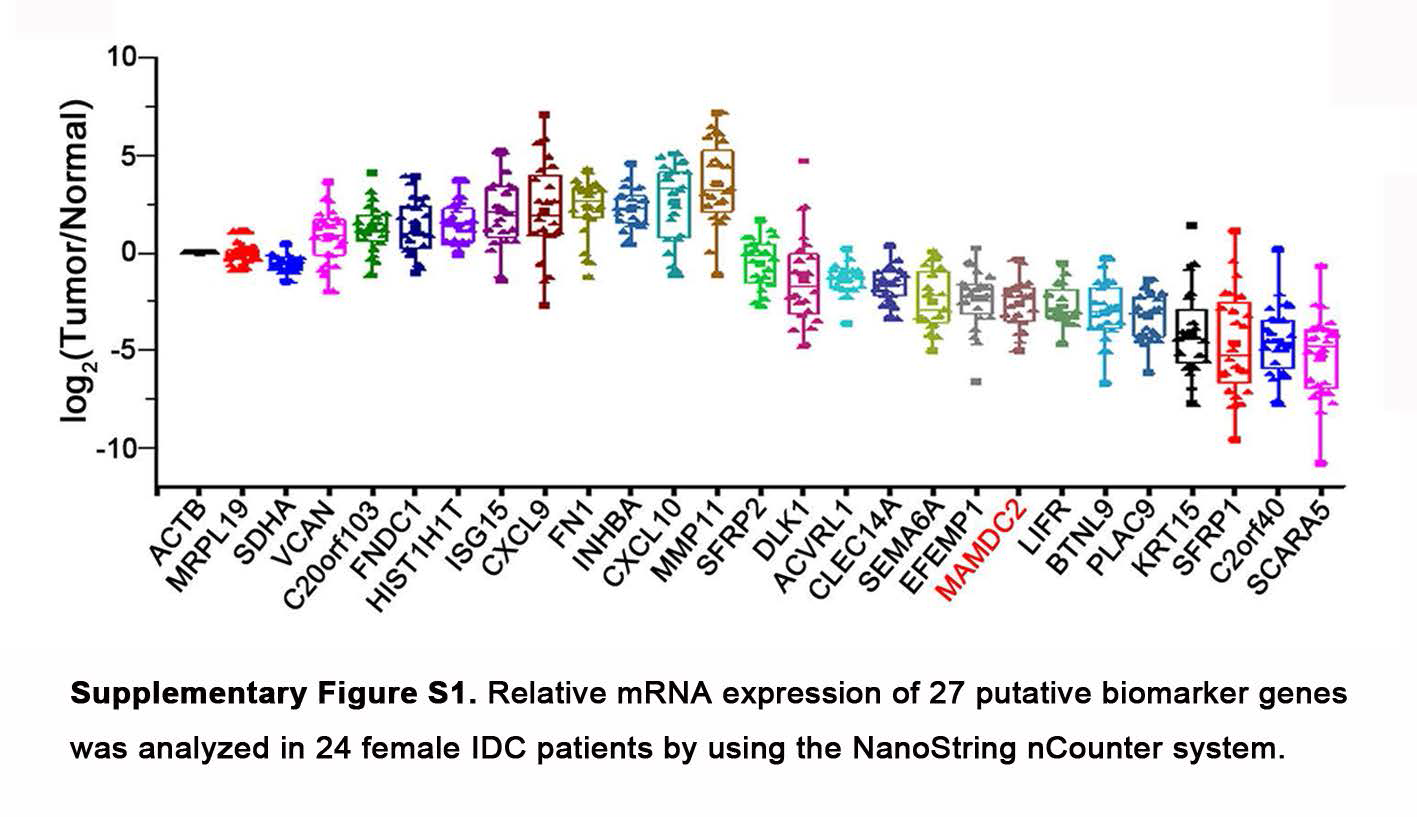

Supplement: Supplementary file 1 — Fig S1 [file CPR-53-e12883-s001.tif]

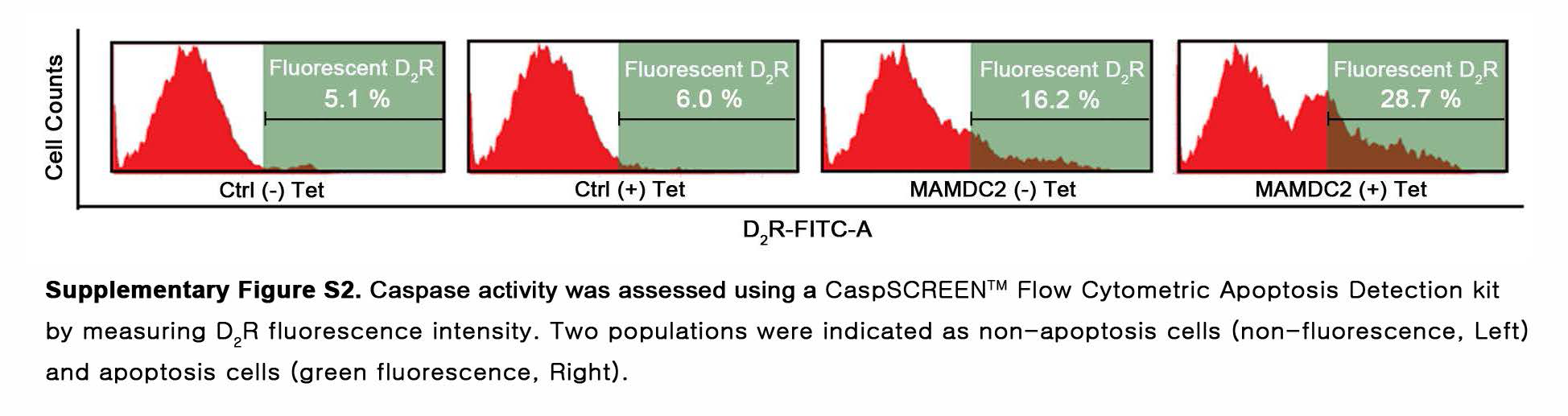

Supplement: Supplementary file 2 — Fig S2 [file CPR-53-e12883-s002.tif]

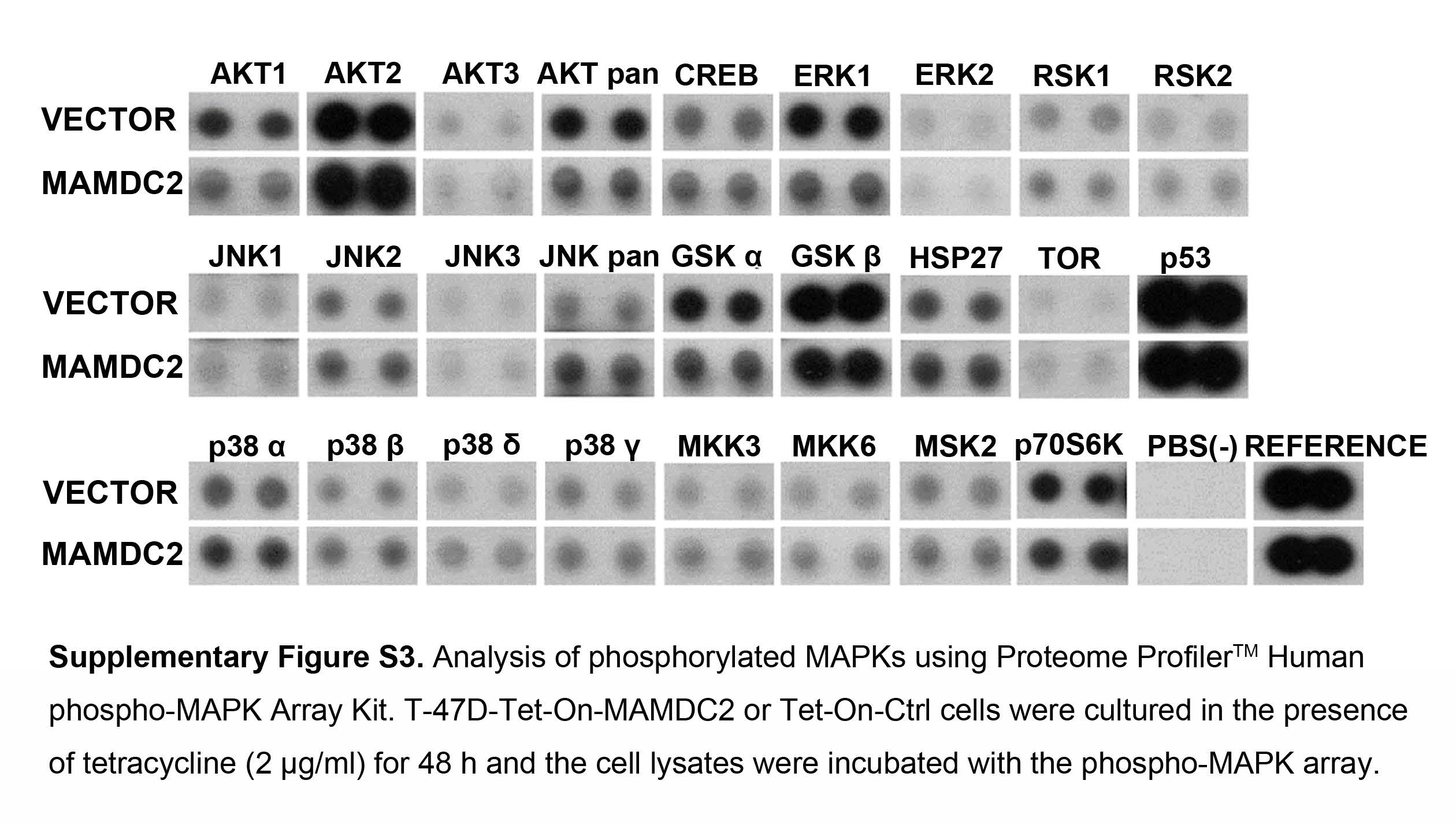

Supplement: Supplementary file 3 — Fig S3 [file CPR-53-e12883-s003.tif]

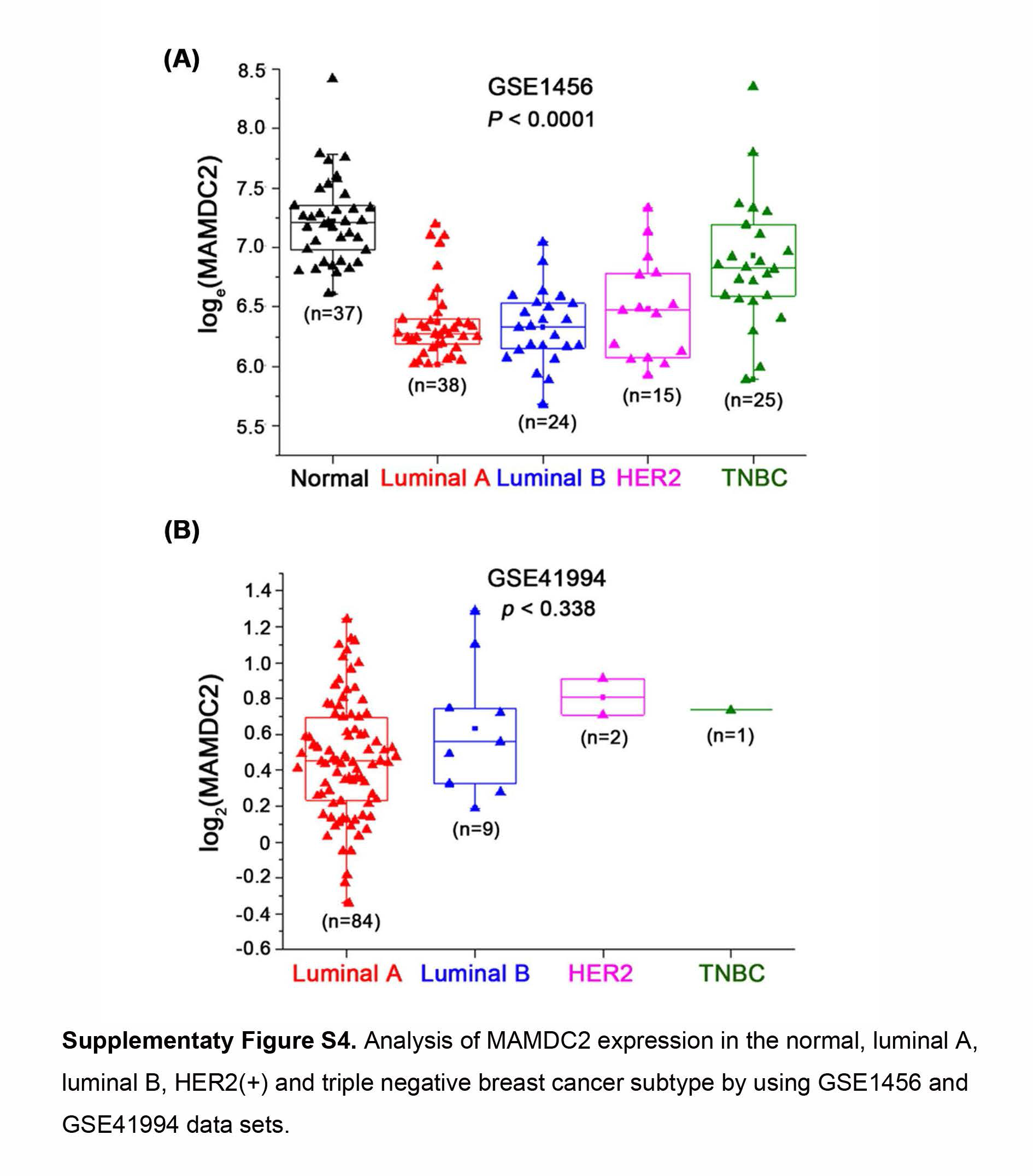

Supplement: Supplementary file 4 — Fig S4 [file CPR-53-e12883-s004.tif]

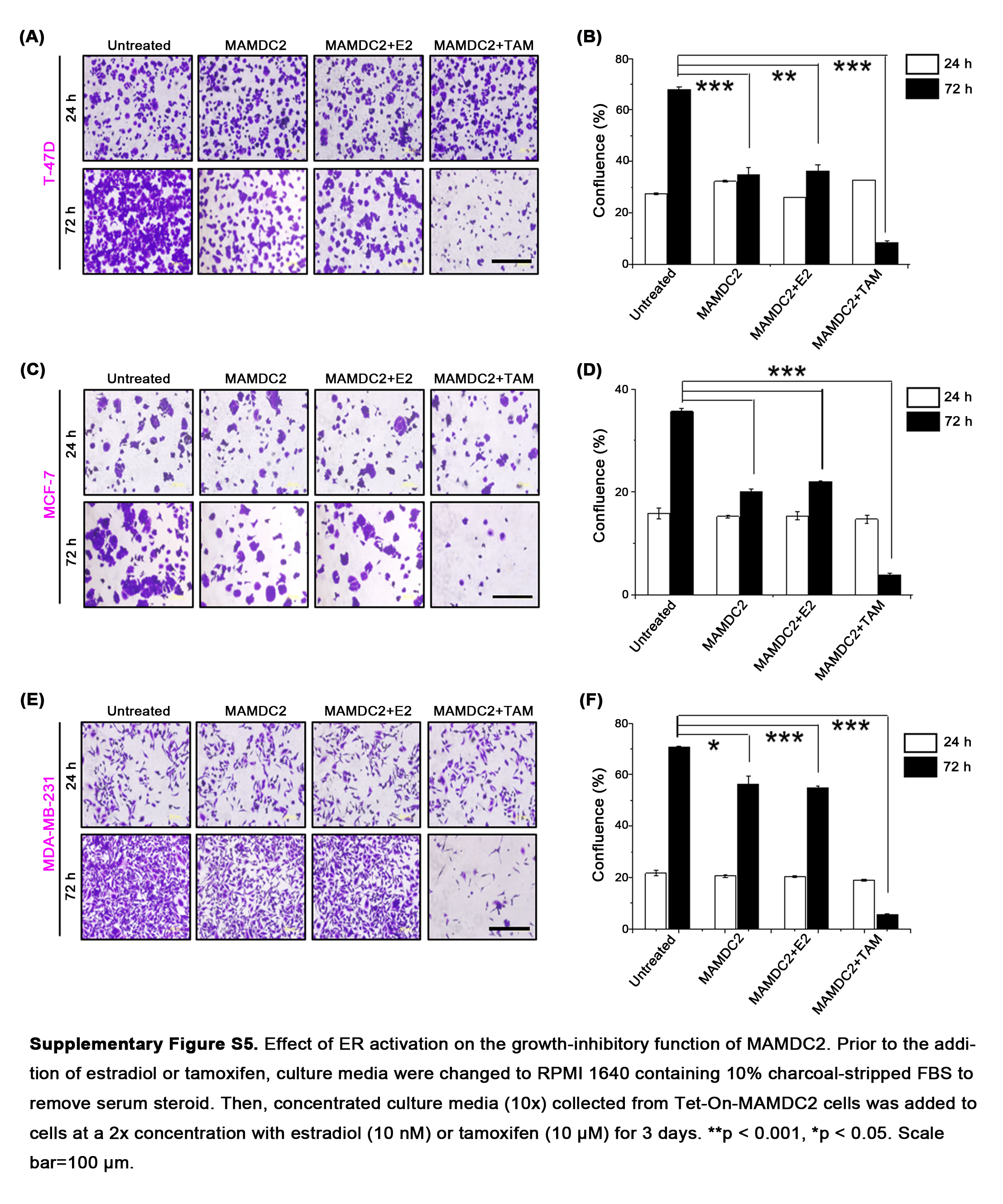

Supplement: Supplementary file 5 — Fig S5 [file CPR-53-e12883-s005.tif]
