# Supplementary material for: MAM domain containing 2 is a potential breast cancer biomarker that exhibits tumour‐suppressive activity
Source: Cell Prolif. 2020 Jul 24;53(9):e12883. doi: 10.1111/cpr.12883 (PMC7507446; doi:10.1111/cpr.12883)
Supplement: Supplementary file 6 — Table S1 [file CPR-53-e12883-s006.doc]

Supplementary Table S1. Patient and biopsy information from the tumors of paired tissue samples.

| **KBN^a^-ID.** | **Age** | **Diagnosis^b^** | **Stage^c^** | **ER^d^** | **PR^d^** | **HER^d^** |
| --- | --- | --- | --- | --- | --- | --- |
| 2036 | 44 | Invasive tubular and cibriform ca | I | **++** | **+** | **-** |
| 1119 | 44 | Invasive tubular and cibriform ca | I | **++** | **+** | **-** |
| 248 | 28 | IDC | IIIA | **++** | **+** | **-** |
| 1858 | 38 | IDC | IIB | **+** | **-** | **-** |
| 2138 | 47 | Microinvasive ductal ca | I | **+** | **-** | **-** |
| 2038 | 47 | IDC | IIA | **+** | **+** | **-** |
| 458 | 55 | IDC | I | **-** | **-** | **-** |
| 1307 | 58 | IDC | IIIC | **+** | **+** | **-** |
| 2190 | 53 | IDC | IIA | **+++** | **+** | **-** |
| 904 | 57 | IDC | IIA | **-** | **-** | **-** |
| 1582 | 48 | IDC | IIIA | **-** | **-** | **-** |
| 1331 | 76 | IDC | IIB | **++** | **-** | **-** |
| 1321 | 36 | Metaplastic ca | IIIA | - | **-** | **-** |
| 1101 | 45 | IDC | IIA | **++** | **+** | **-** |
| 1169 | 56 | IDC | IIA | - | **-** | **-** |
| 501 | 69 | IDC | I | - | **-** | **-** |
| 2232 | 65 | IDC | IIA | **+** | **+** | **-** |
| 1592 | 40 | IDC | IIA | **++** | **+** | **-** |
| 1627 | 27 | Micropapillary ca | IIIC | - | **-** | **-** |
| 2169 | 79 | IDC | IIB | - | **-** | **-** |
| 1525 | 68 | IDC | IIB | **++** | **-** | **-** |
| 2020 | 57 | IDC | IIA | - | **-** | **-** |
| 1673 | 40 | Micropapillary ca | I | - | **-** | **-** |
| 884 | 35 | IDC | IIIA | **+** | **+** | **-** |

^a^KBN; Korea Biobank Network, ^b^IDC; infiltrating duct carcinoma, ^c^Stage ; breast cancer staging on the basis of TMN classification, ^d^ER, PR and HER concentration codes ; - receptor values less than 25%, + receptor values between 25% and 50%, ++ receptor values between 50% and 75%, +++ receptor values more than 75
