# Supplementary material for: MAM domain containing 2 is a potential breast cancer biomarker that exhibits tumour‐suppressive activity
Source: Cell Prolif. 2020 Jul 24;53(9):e12883. doi: 10.1111/cpr.12883 (PMC7507446; doi:10.1111/cpr.12883)
Supplement: Supplementary file 7 — Table S2 [file CPR-53-e12883-s007.doc]

**Supplementary Table S2. Expression analysis of putative breast cancer biomarkers.**

| **Gene** | **Full Name** | **Fold change** |
| --- | --- | --- |
| ACTB (Control) | Actin beta | 1 |
| MRPL19 (Control) | Mitochondrial Ribosomal protein L19 | - 1.04 |
| SDHA (Control) | Succinate dehydrogenase subunit A | -1.43 |
| C20orf103 | Chromosome 20 orf103 | +2.14 |
| CXCL10 | C-X-C motif chemokine 10 | +5.65 |
| CXCL9 | C-X-C motif chemokine 9 | +4.32 |
| FN1 | Fibronectin 1 | +4.40 |
| FNDC1 | Fibronectin type III domain containing 1 | +2.33 |
| HIST1H1T | Histone cluster1 | +2.78 |
| INHBA | Inhibin beta A | +4.44 |
| ISG15 | Interferon-stimulated gene 15 | +4.05 |
| MMP11 | Matrix metalloproteinase 11 | +10.78 |
| VCAN | Versican(ECM proteoglycan) | +1.89 |
| ACVRL1 | Actibin receptor-like kinase 1 | -2.83 |
| BTNL9 | Butyrophillin-like protein | -9.65 |
| C2orf40 | Chromosome 2 orf 40 | -23.58 |
| CLEC14A | C-type lectin family 14 member A | -3.27 |
| EFEMP1 | EGF containing fibulin-like ECM protein 1 | -5.54 |
| KRT15 | Keratin, type 1 cytoskeletal 15 | -16.11 |
| LIFR | Leukemia Inhibitory Factor Receptor | -6.91 |
| MAMDC2 | MAM domain containing 2 | -5.93 |
| PLAC9 | Placenta specific 9 | -10.41 |
| SCARA5 | Scavenger Receptor class A Member5 | -44.01 |
| SEMA6A | Semaphorin 6A | -5.46 |
| SFRP1 | Secreted frizzled-related protein 1 | -23.26 |
| DLK1 | Delta like non-canonical ligand 1 | -1.57 |
| SFRP2 | Secreted frizzled-related protein 2 | -2.28 |
